# Attention maps reveal stimulus-dependent retinal population codes

**DOI:** 10.3389/fncom.2026.1769478

**Published:** 2026-05-15

**Authors:** Francisco Miqueles, Adrián G. Palacios, John Atkinson, María-José Escobar

**Affiliations:** 1 Departamento de Electrónica, Universidad Técnico Federico Santa María, Valparaíso, Chile; 2 Centro Interdisciplinario de Neurociencia de Valparaíso, Facultad de Ciencias, Universidad de Valparaíso, Valparaíso, Chile; 3 Universidad Andrés Bello, Las Condes, Chile

**Keywords:** attention, interpretability, latent representations, neural decoding, retina, retinal ganglion cells, transformers

## Abstract

**Introduction:**

Understanding how deep learning models map neural population activity to stimuli requires both high predictive accuracy and interpretable internal mechanisms.

**Methods:**

In this work, we employ the POYO framework, a scalable transformer architecture based on spike tokenization and latent modeling, to decode large-scale retinal ganglion cell recordings. We ask whether the model's attention mechanisms can provide biologically meaningful insight by evaluating two contrasting conditions: a uniform flash stimulus and a spatiotemporally structured moving ball stimulus.

**Results:**

We show that the model decodes both stimuli reliably and adapts rapidly to new preparations via fine-tuning, suggesting the capture of transferable population codes. We then analyze the model's internal organization, revealing that encoder attention patterns adapt to stimulus complexity: attention heads appear synchronized and broadly distributed for the flash stimulus, whereas they exhibit heterogeneous, specialized allocation strategies for the moving ball. By aggregating attention weights to identify the most relevant neurons for each task, we demonstrate that these high-attention units possess distinct physiological signatures—concentrating sustained, high-firing rates responses for the flash vs. diverse kinetics for the structured input. We confirm the causal validity of these findings via attention-guided ablations, where the progressive removal of these top-ranked units yields systematic losses in decoding performance. Furthermore, we expand the analysis to the decoder's attention, uncovering stimulus-specific retrieval strategies where individual heads exhibit distinct directional tuning preferences.

**Discussion:**

We conclude that generic attention mechanisms can spontaneously recover biological coding strategies, identifying functionally distinct neural subpopulations without supervision, thus validating the utility of transformer-based architectures for neuroscientific discovery.

## 1 Introduction

The retina is a highly organized neural tissue located at the back of the eye, responsible for capturing incoming light and converting it into electrical signals that can be interpreted -through a dynamic learning processes mechanism- by the brain. This process begins when photoreceptors absorb photons and, through interactions with inter-neurons and retinal ganglion cells (RGCs), generate sequences or trains of action potentials that travel via the optic nerve to higher visual centers (Dowling, 2012; Kolb et al., 1995). Understanding how these signals encode the diverse visual stimuli found in nature is fundamental to revealing the mechanisms by which sensory input is transformed into perception. A natural counterpart to this question is whether one can invert the process: given the neural responses, can the original stimulus be reconstructed? This challenge defines the problem of neural decoding, that seeks to reconstruct or infer a stimulus from the electrical responses of a population of neurons. In the visual system, this involves estimating features such as shape, motion, or contrast of the original stimulus from the recorded spiking activity of RGCs (Warland et al., 1997; Marre et al., 2015; Rieke et al., 1999). Precise decoding models provide a dual benefit: they help determine how information about the external world is preserved in neural activity, and they aid in the design of visual prostheses and brain-computer interfaces.

Early decoding approaches relied on linear models, valued for their simplicity and direct interpretability. These methods allowed clear links between model parameters and physiological features, helping to identify the contribution of specific neurons or receptive field (RF) properties to stimulus reconstruction (Warland et al., 1997; Nichols et al., 2013). As datasets became richer and stimuli more complex, however, linear approaches proved to be insufficient. To capture nonlinear dependencies, researchers turned to artificial neural networks (ANN), Bayesian decoders, and later deep learning architectures, which achieved markedly higher accuracies in decoding natural images and dynamic visual scenes (Parthasarathy et al., 2017; Botella-Soler et al., 2018; Yu et al., 2025).

More recently, transformer-based architectures have reshaped the decoding landscape. Building on advances in language (Vaswani et al., 2017) and vision (Dosovitskiy et al., 2021), they provide scalable representations and excel at capturing long-range dependencies. This attention-based mechanism offers an inherent path toward interpretability; by quantifying how a model dynamically prioritizes specific input features, researchers can move beyond black-box predictions to uncover the underlying structure of neural data. The POYO framework exemplifies this progress by introducing a unified tokenization scheme and leveraging PerceiverIO-style attention to decode neural activity across sessions, animals, and tasks with unprecedented robustness (Azabou et al., 2023). A key distinction of this approach is that, unlike traditional decoders that rely on binarizing spike trains into discrete time bins, POYO processes individual spikes as continuous-time events. This point-process framework allows the model to preserve the sub-millisecond precision of the retinal output, which is fundamental for capturing the rapid neural dynamics triggered by moving stimuli.

Its extension, POYO+, further scaled this idea, showing that training on highly diverse datasets spanning cell types, brain regions, and behaviors not only improves decoding performance but also enables cross-task and cross-region transfer (Azabou et al., 2025). Importantly, the authors explored the interpretability of their model by analyzing the latent space trained on large-scale datasets, such as the Allen Brain Observatory. They observed that, even without explicit supervision, the learned unit embeddings spontaneously organized into clusters corresponding to distinct anatomical brain regions and cellular sub-types. While this provides promising evidence that large-scale models can capture biologically meaningful patterns, the analysis remains largely descriptive and does not yet yield strong physiological conclusions.

POYO capability raises a deeper question: Can the internal mechanisms of these high performing models, specifically their attention and latent representations, be used to reverse engineer the computational strategies of the neural population itself? In this work, we test the hypothesis that transformer-based decoders can reveal distinct population coding regimes. We propose that the attention mechanism, which dynamically weights the contribution of different inputs, provides a direct readout of how the neural population allocates its computational resources. We trained the POYO model on large-scale RGC recordings under two contrasting stimuli: a uniform flash and a complex moving ball. Beyond benchmarking accuracy, we analyzed whether the model's internal dynamics would expose a shift from a concentrated, specialized code for the simple, global stimulus to a distributed, diverse representation for the complex, spatiotemporal one. Our goal was to determine if interpretable artificial intelligence can move beyond performance and generate testable hypotheses about adaptive neural computation.

The manuscript is organized as follows. Section 2 details the electrophysiological procedures, light stimuli, and the POYO model architecture, along with the methodology for attention analysis. Section 3 presents the decoding performance benchmarks and the analysis of encoder and decoder attention patterns in relation to physiological properties. Section 4 discusses the implications of our findings for interpretable neural decoding and addresses current limitations. Finally, Section 5 summarizes the main conclusions and future research directions.

## 2 Materials and methods

### 2.1 Electrophysiological recordings and spike sorting

We followed the complete electrophysiological recording protocol described in Ravello et al. (2019); Escobar et al. (2018). In brief, we used a multielectrode array (MEA) (USB256, Multichannel Systems GmbH, Reutlingen, Germany) with 252 electrodes and a sampling rate of 20 kHz to record RGCs in isolated retinal tissue. We stored all recordings on a computer for offline analysis. We obtained retinal tissue from 5 adult wild-type mice (3 males and 2 females, 3 months old). Before each experiment, we dark-adapted the animals for 30 min. Subsequently, animals were deeply anesthetized via inhalation of 2.5% isoflurane (Baxter, Deerfield, IL, USA) dissolved in *O*_2_ (flow rate 300 mL/min in a 3L chamber) using a small animal anesthesia system (RWD Life Science Co., China), and immediately euthanized by decapitation. We quickly enucleated the eyes under dim red light and immersed the eyecups in Ames medium with bicarbonate buffer (Sigma-Aldrich, St. Louis, MO, USA) at 32°C and pH 7.4, continuously oxygenated with 95% (*O*_2_) and 5% (*CO*_2_). We gently separated small pieces of retina from the retinal epithelium and placed them on a rod device supporting a dialysis membrane ring (MWCO-25000, Spectrumlabs, Rancho Dominguez, CA, USA), which we treated with polylysine (Product P4707, Sigma-Aldrich, St. Louis, MO, USA) to facilitate contact between the RGC side of the retina and the surface of the MEA electrodes.

We performed spike detection and classification using SpyKING Circus (Pierre Yger & Olivier Marre, Institut de la Vision, Paris, France) and SpikeInterface software (SpikeInterface Development Team (Open Source), Lausanne, Switzerland) (Yger et al., 2018). We retained units for further analysis only if their interspike interval (ISI) violation rate was less than 1.5%, their signal-to-noise ratio (SNR) exceeded 3 standard deviations, and they exhibited at least five trials with 10 or more spikes within the first 10 trials. This method ensures the reliability of unit classifications by stringent criteria. As responsiveness varied between the two visual protocols (detailed in Section 2.2), we obtained distinct unit counts for each. For the flash stimulus, we identified 512, 446, and 339 units for the males, and 373 and 339 for the females. For the moving ball stimulus, we recovered 490, 400, and 314 units for the males, and 377 and 366 for the females.

### 2.2 Light stimuli

We generated and delivered light stimuli using MATLAB software (The MathWorks, Inc., Natick, MA, USA) and Psychtoolbox [Mario Kleiner et al. (Open Source), Tübingen, Germany] via a standard LED projector (PB 60G-JE, LG). We projected the images through a custom optical bench aligned with the photoreceptor layer. We calibrated spectral emissions at 460 and 520 nm using a USB4000 spectrophotometer (Ocean Optics Inc., Orlando, FL, USA) and measured the optical power at the sample plane using a Newport 1918-R optical power meter, yielding an average irradiance of 70 nW/mm^2^. Finally, we displayed 400 × 400 pixel images spanning a 2 × 2 mm area on the MEA array under an inverted microscope [Eclipse T200 (Nikon Corporation, Tokyo, Japan), Nikon (Nikon Corporation, Tokyo, Japan)], where each pixel represented approximately 4 microns.

The stimulation protocol included two sets of visual stimuli. The first stimulus consisted of full-field flashes used to measure global luminance responses. The protocol comprised a 3-s flash ON followed by a 3-s flash OFF. We repeated this sequence for 10 trials.

The second stimulus consisted of a moving ball displayed against a black background. The ball was a cyan ellipsoid with a mean pixel intensity of 154.2 (scale 0–255). The ball subtended approximately 28.0 × 36.6 pixels throughout the presentation. We presented the stimulus for 20 s and repeated it 10 times. To ensure an irregular, persistent trajectory that activated different retinal regions, we generated the motion using fractional Brownian motion with a Hurst exponent of *H* = 0.9. The ball moved with a mean speed of 232 pixels/second.

### 2.3 Light features

To characterize the light-evoked response properties of each RGC, we computed several indices: polarity, sustain index, and latency. We estimated a flash bias response to classify each RGC as ON, OFF, or ON-OFF based on their relative amplitudes, calculated as:


$$\begin{array}{l} f_{BR}=\frac{f_{ON}-f_{OFF}}{f_{ON}+f_{OFF}} \end{array}$$

where *f*_*ON*_ and *f*_*OFF*_ are the maximum spike counts during the ON or OFF parts of the flash stimuli. A value of 1 corresponds to a pure ON unit, a value of -1 corresponds to a pure OFF unit, and 0 corresponds to a pure ON-OFF unit.

We calculated a sustain index (*S*_*i*_) to evaluate the temporal profile of each response (sustained vs. transient):


$$\begin{array}{l} S_{i}=\frac{f_{ON/OFF}-\bar{f}}{f_{ON/OFF}+\bar{f}} \end{array}$$

where $\bar{f}$ represents the mean firing rate during the entire flash sequence and *f*_*ON*/*OFF*_ is the total spike count during the ON or OFF parts of the stimuli. A sustain index of 1 corresponds to a pure transient response, while a value of 0 corresponds to a pure sustained response. Finally, we defined the flash latency as the time interval between stimulus onset (either ON or OFF phase) and the peak firing response. We also computed the mean firing rate for each unit as the total number of spikes divided by the total duration of the flash stimulus.

### 2.4 Model architecture

**Figure 1:**
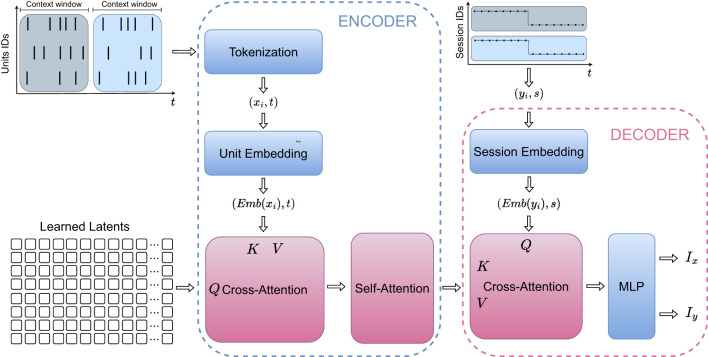
Schematic of the POYO architecture adapted for retinal decoding. The diagram illustrates the information flow. Input spikes **(Left)** represent the neural activity, which the cross-attention mechanism compresses into a fixed set of learned latents (center). Self-attention blocks process these latents, and the final stage queries them to predict the temporal stimulus features **(Right)**.

We employed the POYO framework (Azabou et al., 2023), a transformer-based architecture designed to learn robust representations of neural population dynamics. Unlike standard approaches that bin neural activity, we utilized POYO's spike-based tokenization strategy, where the model input comprises tokens representing individual retinal spike events within 1-s context windows. We organized the processing pipeline into two functional components: an encoder and a decoder. The information flow of the model is illustrated in Figure 1.

In the encoder we mapped the high-dimensional input spike tokens onto a fixed set of latent tokens using a cross-attention mechanism. This step effectively reduces dimensionality while preserving relevant contextual features. Subsequently, we applied a self-attention module to the latent tokens, enabling the model to capture long-range temporal dependencies and interactions across neuronal units.

In the decoder we projected the refined latent representations onto output tokens through an additional cross-attention operation. We then integrate these output tokens with session-level embeddings: learnable vector representations that capture the specific characteristics of each experimental session. This mechanism accounts for variability across different retinal preparations and recording days, enabling a unified decoding strategy for multiple datasets. Finally, we passed the output tokens through a multi-layer perceptron (MLP) (David E. Rumelhart, 1986), which transforms these high-level representations into continuous-valued predictions corresponding to the stimulus features. For the flash stimulus, which consists of full-field luminance variations without spatial structure, we condensed each frame into a single scalar value to form the temporal stimulus curve. For the moving ball stimulus, we used both the *x* and *y* positional coordinates of the ball as output signals.

### 2.5 Model training and implementation details

To ensure reproducibility, we partitioned the dataset into training (70%), validation (10%), and test (20%) sets using non-overlapping temporal blocks. We implemented the architecture using the POYO framework with a latent dimension of 128, a latent sequence length of 64, and a depth of six layers. Both cross-attention and self-attention mechanisms utilized eight heads each, with a head dimension of 64. To prevent overfitting, we applied a dropout rate of 0.3 across the feed-forward, linear, and attention layers. We trained the model to minimize the Mean Squared Error (MSE) between the reconstructed and ground-truth stimuli using the AdamW optimizer (initial learning rate 1 × 10^−4^, weight decay 1 × 10^−2^). Training lasted 100 epochs for single experiments and 400 for multiple experiments, with a batch size of 16, employing a scheduler that decayed the learning rate by a factor of 0.1 during the final 25% of the epochs.

### 2.6 Analysis of encoder attention

To quantify the contribution of individual retinal units, we analyze the cross-attention mechanism in the encoder. For a given context window *i* containing *N*_*i*_ spike tokens, the model computes an attention matrix **A**_*h, i*_ for each head *h*∈{1, …, *H*}. These matrices are defined as:


$$\begin{array}{l} {\text{A}}_{h,i}\in {\mathbb{R}}^{L\times N_{i}} \end{array}$$

where *L* is the fixed number of latent tokens. Each element $a_{j,k}^{h,i}$ represents the attention weight assigned by the *j*-th latent token to the *k*-th spike event for the *h*-th head. To obtain a spike-specific relevance score that is independent of the latent projection, we collapse the latent dimension by averaging the weights across all *L* tokens:


$$\begin{array}{l} {\bar{w}}_{h,i,k}=\frac{1}{L}\overset{L}{\underset{j=1}{\sum }}a_{j,k}^{h,i} \end{array}$$

To ensure that the importance scores are comparable across windows with varying spike densities and to prevent bias from high-firing intervals, we normalize the weights by the total spike count within the window:


$$\begin{array}{l} {ŵ}_{h,i,k}={\bar{w}}_{h,i,k}\cdot N_{i} \end{array}$$

The resulting normalized weights ŵ_*h, i, k*_ are used to color-code raster plots, where each spike *k* from unit *u* is plotted at its occurrence time *t*_*k*_ with intensity ŵ. This procedure allows for an objective comparison of how different heads prioritize specific neural subpopulations across different visual stimuli.

#### 2.6.1 Entropy and statistical divergence

To quantify the temporal organization and focus of attention, we compute the Shannon entropy *E* for each attention head *h* within every 1-s context window *i*. Using the spike weights ${\bar{w}}_{h,i,k}$, the entropy (in nats) is defined as:


$$\begin{array}{l} E\left(h,i\right)=-\overset{N_{i}}{\underset{k=1}{\sum }}{\bar{w}}_{h,i,k}\ln\left({\bar{w}}_{h,i,k}\right) \end{array}$$

This transformation yields a time-varying metric with a resolution of 1 Hz, where each value *E*(*h, i*) is assigned to the timestamp corresponding to the end of its respective window. To determine whether individual attention heads developed specialized functional roles, we performed pairwise two-sample Kolmogorov-Smirnov (K-S) tests across all head combinations for both stimuli. This non-parametric test allowed us to assess significant differences in the cumulative distribution of entropy values, providing a robust measure of head divergence.

#### 2.6.2 Causal relevance and unit ranking

To assess the functional contribution of individual retinal units *u* to decoding performance, we define an importance score *I*(*u*) based on the average attention weight assigned to its spikes. This score is calculated by averaging the spike-specific relevance scores across all attention heads and all spikes belonging to unit *u*:


$$\begin{array}{l} I\left(u\right)=\frac{1}{HWN_{i}^{u}}\underset{h\in H}{\sum }\underset{i\in W}{\sum }\underset{k\in N_{i}^{u}}{\sum }{\bar{w}}_{h,i,k} \end{array}$$

where *W* is the number of context windows with in the stimulus duration and $N_{i}^{u}$ is the number of spikes of the unit *u* in the context window *i*. This metric identifies the neurons that the model consistently considers most relevant for the decoding task, independent of their total firing rate.

To validate the predictive relevance of this ranking, we performed a causal ablation analysis by comparing the decoding degradation under two exclusion strategies:

- Attention-guided ablation: Units are removed sequentially in descending order of *I*(*u*).
- Random control: Units are removed in a stochastic order to establish a performance baseline.

For each removal step, we re-evaluated the model on the test set and computed the coefficient of determination *R*^2^ to track the degradation in decoding integrity. For each stimulus type, we repeated this procedure for eight independent training runs (seeds).

#### 2.6.3 Physiological characterization of high-attention units

We further examined whether the units prioritized by the encoder share distinctive physiological properties. For each stimulus and each seed, we determined the number of units (*K*) whose cumulative removal reduced *R*^2^ to 0.5 in the ablation curve, producing a top-*K* list per seed. To obtain a stimulus-specific set of consistently attended units, we aggregated the eight lists and retained only those units that appeared in at least half of the seeds. We then compared the distributions of light-response features between these consensus subsets and the global population of recorded units. To evaluate these differences, we employed a non-parametric statistical K-S test to detect differences in the overall shape and cumulative distribution of the features.

### 2.7 Analysis of decoder attention

To reconstruct the visual stimulus, the decoder employs a cross-attention mechanism that selectively retrieves information from the learned latent representations. For a given reconstruction sequence, the model computes a decoder attention matrix **D**_*h*_ for each head *h*. Unlike the encoder, which maps spikes to latents, the decoder operates on the output temporal grid:


$$\begin{array}{l} {\text{D}}_{h}\in {\mathbb{R}}^{M\times L} \end{array}$$

where *M* is the number of output tokens (timestamps). Each element $d_{m,j}^{h}$ in the matrix represents the attention weight that the *m*-th output token assigns to the *j*-th latent vector. We visualize these matrices as heatmaps to observe the temporal dynamics of information integration.

#### 2.7.1 Stimulus-attention coupling analysis

To quantify the functional link between internal attention dynamics and stimulus reconstruction, we calculate the Pearson correlation coefficient *r* between the decoder entropy traces and the relevant external dynamics. The decoder entropy *E*(*h, m*), of the *h*th head, measures the uncertainty in latent selection for each output token *m*. Using the weights $d_{m,j}^{h}$ from the decoder attention matrix **D**_*h*_, the entropy is defined as:


$$\begin{array}{l} E\left(h,m\right)=-\overset{L}{\underset{j=1}{\sum }}d_{m,j}^{h}\ln\left(d_{m,j}^{h}\right) \end{array}$$

This metric is computed at the sampling frequency of the reconstructed stimulus to capture high-resolution temporal dynamics. The correlation strategy then adapts to the specific structure of each task:

- Flash: We compute a single correlation value for each head by comparing its entropy trace directly with the light intensity profile.
- Moving ball: To account for spatial complexity, we decompose the trajectory into rectified Cartesian components (+*X*, −*X*, +*Y*, −*Y*). For each attention head, we construct two directional correlation vectors: one representing positive displacements (*X, Y*) and another for negative displacements (−*X*, −*Y*).

### 2.8 Comparative decoding architectures

To evaluate the specific advantages of the POYO framework and address the performance benchmarks of contemporary machine learning tools, we implemented four baseline architectures representing distinct decoding strategies:

- Optimal Linear Estimator (OLE): We implemented a Ridge regression model to establish a fundamental linear decoding baseline (Warland et al., 1997). This model maps binned firing rates directly to stimulus coordinates. We utilized a regularization parameter α = 0.1, determined via cross-validation, to ensure a robust linear estimation while preventing overfitting.
- Neural Data Transformer 2 (NDT2): We adapted the NDT2 architecture to process binned neural activity as a representative of modern transformer-based decoders (Ye and Pandarinath, 2021). The model features a 4-layer Transformer encoder with 8 attention heads, a hidden dimension of 128, and a feed-forward dimension of 512. We integrated learnable positional encodings to preserve the sequential dependencies of the binned inputs.
- CEBRA: We employed the CEBRA framework to perform hypothesis-driven latent space exploration (Schneider et al., 2023). We configured the model with an offset10-model architecture and optimized it using a contrastive InfoNCE loss. To quantify its decoding capabilities, we mapped the resulting 3D latent embeddings to the stimulus space using a k-Nearest Neighbors (kNN) decoder.
- Population State-Space Model (POSSM): We implemented a state-space approach using Gaussian Process Factor Analysis (GPFA) to capture underlying population dynamics (Yu et al., 2009). We extracted a 32-dimensional latent state representing the neural manifold and subsequently applied a Ridge regressor to decode stimulus features.

These benchmarks provide a comprehensive landscape to situate the performance of spike-token attention mechanism against both classical linear methods and modern latent-modeling frameworks.

## 3 Results

### 3.1 Decoding performance and adaptation across retinal preparations

Before addressing the internal mechanisms of the model, we verified the decoding performance across different retinal pieces. We evaluated neural recordings from five individual retinas under two conditions: independent training/testing on a single retina and a fine-tuning setup, in which we pre-trained the model on four retinas and subsequently adapted it to a fifth.

First, in Table 1, we compare the decoding accuracy of the POYO framework against several established machine learning baselines for both stimulus types. For the moving ball stimulus, the POYO model achieves an *R*^2^ of 0.978, whereas the linear OLE baseline yields 0.749 and the state-space POSSM reaches 0.690. When evaluating the flash stimulus, we observe that POYO maintains a high performance (*R*^2^ = 0.994), contrasting with the lower accuracy obtained by the binned NDT2 transformer (*R*^2^ = 0.844) and the contrastive CEBRA model (*R*^2^ = 0.821). These measurements characterize a consistent performance gap between the spike-token attention mechanism and methods relying on traditional binned firing rates or linear approximations.

**Table 1:** Benchmark of decoding performance (*R*^2^) across different machine learning architectures for both visual stimuli (Flash and Moving Ball).

Architecture	Decoding strategy	Flash (*R*^2^)	Moving ball (*R*^2^)
OLE	Optimal linear estimator	0.327	0.749
POSSM	State-space model	0.426	0.690
CEBRA	Contrastive learning	0.821	0.744
NDT2	Binned transformer	0.844	0.975
POYO	**Spike-token attention**	**0.994**	**0.978**

The comparison includes traditional linear baselines and state-of-the-art neural decoders. Values in bold indicate the architecture with the highest *R*^2^ score for each decoding task.

**Figure 2:**
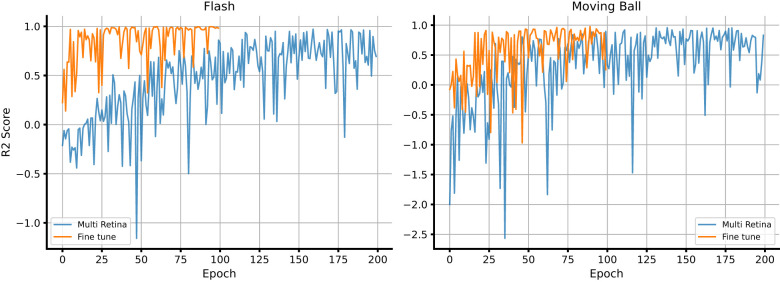
Comparison of learning curves between fine-tuning and training from scratch. The panels display the training trajectories for both stimuli [**(Left)**: flash; **(Right)**: moving ball], comparing the multi-retina pre-trained model against the fine-tuning process on a held-out retina. The pre-trained model requires only a few epochs of fine-tuning to match the performance of training from scratch, illustrating the presence of transferable representations that allow efficient adaptation to new preparations.

Table 2 summarizes the decoding accuracy for both stimulus types across all experimental sessions. For the flash stimulus we observed that the model consistently reached *R*^2^ values above 0.98, reflecting near-perfect reconstruction of the stimulus dynamics. For the moving ball stimulus we found that performance remained high across retinas, with most experiments yielding *R*^2^ values between 0.97 and 0.98. We further examined the model's capacity to generalize to unseen retinal recordings using the fine-tuning configuration. As illustrated in Figure 2, we observed that the pre-trained multi-retina model required only a few epochs of fine-tuning to converge on a new retina, reaching decoding accuracy comparable to that obtained by training from scratch. This rapid convergence indicates that the pre-trained encoder-decoder retains transferable representations of retinal population dynamics, facilitating efficient adaptation without extensive retraining.

**Table 2:** Performance comparison across experiments for two visual stimuli: flash and moving ball.

Experiment	Flash			Moving ball		
	*R* ^2^	Units	Spikes	*R* ^2^	Units	Spikes
1st	0.994	512	219,908	0.978	490	644,050
2nd	0.997	446	313,084	0.971	400	557,731
3rd	0.993	399	270,732	0.974	377	656,751
4th	0.989	373	234,452	0.983	366	487,480
All	0.987	1,730	1,038,176	0.972	1,633	2,346,012
5th (fine-tuned)	0.995	339	197,143	0.889	314	348,988

Metrics include coefficient of determination (*R*^2^), number of units, and total spikes.

### 3.2 Encoder attention allocation strategies under different stimuli

We next examined how the encoder distributes attention across retinal spikes when mapping inputs to the latent space. For each context window, we computed attention scores between latent tokens and individual spike tokens and normalized these values to allow fair comparison across varying spike counts.

**Figure 3:**
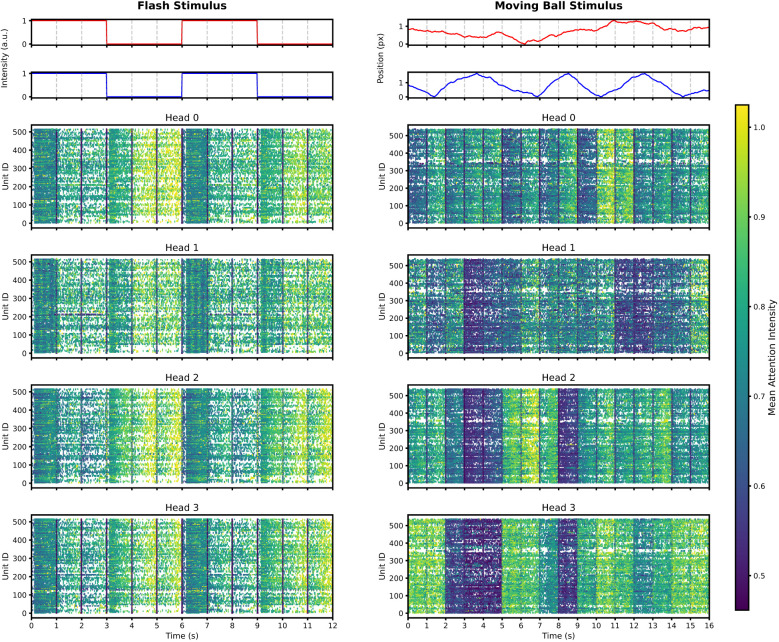
Visualization of encoder attention allocation during flash and moving ball stimuli. The raster plots display retinal spike activity color-coded by the normalized attention weights, averaged across the latent dimension for a representative subset of four attention heads per stimulus. **(Left)** Under the uniform flash stimulus, attention patterns appear temporally synchronized across heads, consistently prioritizing stimulus intervals aligned with global luminance changes. **(Right)** In contrast, during the moving ball stimulus, attention patterns exhibit higher heterogeneity across heads and time, with high-attention bands aligning with rapid changes in the spatiotemporal dynamics of the trajectory. The top panels for each column illustrate the ground-truth stimulus features: light intensity for the flash **(Left)** and the *x* and *y* positional coordinates for the moving ball **(Right)**.

Figure 3 displays raster plots where spike activity is color-coded by the normalized attention weights across all latents for a representative subset of four attention heads per stimulus. Under the uniform flash stimulus, we observed a broadly distributed attention pattern that lacks clear functional boundaries, reflecting the global temporal drive of the light intensity changes. By contrast, the moving ball stimulus triggers a more selective allocation strategy, where individual heads emphasize specific neuronal subsets and discrete time intervals that align with rapid changes in the trajectory.

**Figure 4:**
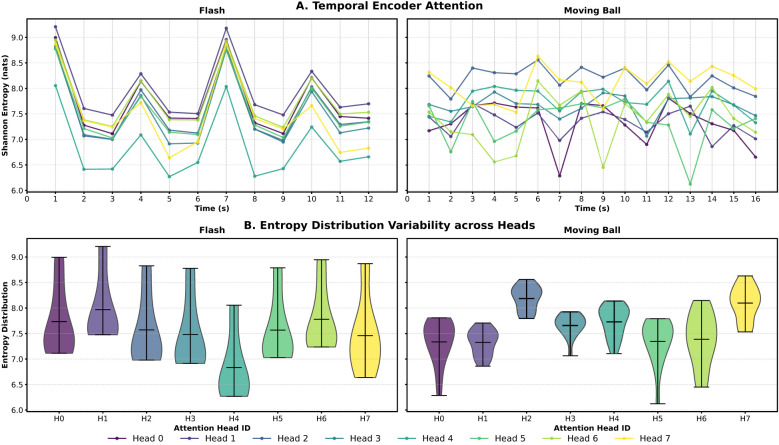
Statistical analysis of encoder attention entropy. **(A)** Temporal evolution of Shannon Entropy for all attention heads during the flash (left) and moving ball (right) stimuli. In the flash condition, entropy traces show high synchronization and sharp drops aligned with global luminance changes. In contrast, the moving ball stimulus induces heterogeneous entropy profiles, reflecting specialized attention allocation across heads. **(B)** Distribution of entropy values across individual heads. The violin plots illustrate the variability and range of attention focus, where diverse distributions in the moving ball condition highlight functional specialization among heads compared to the more uniform behavior observed during the flash stimulus.

To move beyond these qualitative observations, we analyzed the temporal organization of these patterns through the Shannon entropy of attention distributions. As shown in Figure 4A, we analyzed the temporal evolution of attention entropy sampled at 1-s intervals. For the flash stimulus, the entropy traces across all heads exhibit high temporal synchronization, with sharp, simultaneous drops in entropy corresponding to major luminance transitions. This indicates that the encoder adopts a unified pooling strategy when processing global changes. Conversely, the moving ball stimulus elicits highly heterogeneous entropy profiles, where different heads independently adjust their focus to capture specific spatiotemporal features of the motion. The increased variability in entropy distributions, illustrated by the violin plots in Figure 4B, suggests that stimulus complexity forces the architecture to diversify its internal focus.

We validated these observations through a pairwise statistical comparison summarized in Table 3. By consolidating the results into a dual-diagonal format, we contrasted the head specialization between both experimental conditions: the lower diagonal represents the flash stimulus, while the upper diagonal shows the moving ball results. The K-S test reveals that while a substantial portion of head pairs maintain redundant distributions under the flash stimulus (*P*>0.05), the moving ball stimulus induces a profound functional divergence. Nearly all comparisons for the complex motion reached extreme significance levels (e.g., *P* < 10^−9^ for Head 2). This marked difference in both the scale and frequency of statistical significance confirms that while global stimuli maintain a largely redundant processing regime, structured motion triggers a highly specialized and heterogeneous recruitment of attention heads.

**Table 3:** Pairwise comparison of attention head entropy distributions.

		Heads							
		H0	H1	H2	H3	H4	H5	H6	H7
Heads	H0	–	0.426	**1.06e-07**	0.093	**0.035**	1.00	0.716	**1.20e-04**
	H1	**0.008**	–	**3.33e-09**	**0.001**	**0.003**	0.215	0.426	**1.65e-05**
	H2	0.536	**0.008**	–	**1.65e-05**	**0.003**	**3.33e-09**	**1.20e-04**	0.716
	H3	0.099	**0.008**	0.869	–	**0.035**	0.093	0.093	**1.20e-04**
	H4	**0.001**	**2.04e-04**	**0.008**	**0.008**	–	**0.035**	0.215	**0.003**
	H5	0.536	**0.008**	0.998	0.536	**0.008**	–	0.716	**1.20e-04**
	H6	0.869	0.099	0.099	**0.008**	**0.001**	0.099	–	**0.003**
	H7	0.536	**0.008**	0.536	0.869	**0.031**	0.536	0.256	–

This matrix combines p-values from two-sample Kolmogorov-Smirnov tests. The upper diagonal (blue) displays results for the moving ball stimulus, while the lower diagonal (orange) corresponds to the flash stimulus. Values in bold indicate *P* < 0.05.

### 3.3 Causal relevance and physiological properties of high-attention units

To determine whether the attention assigned by the encoder reflects functionally important neurons, we examined the overlap among the top *K* attended units across eight independent training seeds per stimulus. We observed that several units were consistently selected across runs, indicating stable attention patterns and suggesting that the model repeatedly identifies a core subset of neurons critical for decoding. We then assessed how decoding performance changed as we systematically removed these high-attention units compared to a random removal baseline.

**Figure 5:**
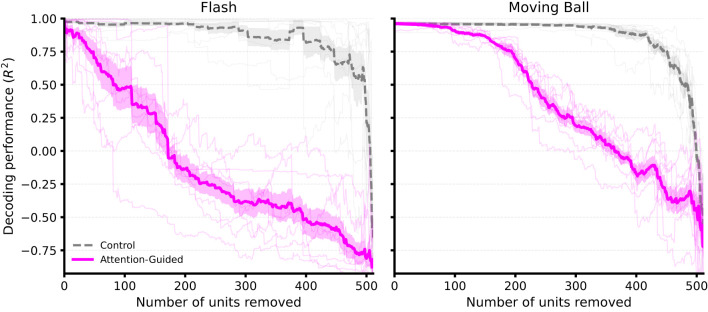
Impact of progressive ablation of high-attention units on decoding performance. Each panel displays the *R*^2^ scores as a function of the number of units progressively removed from the input population for the flash **(Left)** and moving ball **(Right)** stimuli. The solid magenta curves illustrate the attention-guided ablation, where units are removed in descending order of their cumulative encoder attention weights averaged across independent seeds (*n* = 8). The dashed gray curves represent the random control, where units are removed in a stochastic order, with the surrounding shaded gray area indicating the variability across multiple iterations. For both stimuli, the performance decline is significantly more rapid under attention-guided removal compared to the control, confirming that the attention mechanism effectively prioritizes neurons with high predictive relevance for stimulus reconstruction.

Figure 5 shows the *R*^2^ values as a function of the number of removed units for both the attention-guided strategy and the random control. For both stimuli, we found that performance decreased sharply when we excluded the most-attended units, whereas the random removal of units resulted in a significantly slower decay in decoding quality. This gap confirms that attention weights accurately capture neurons with high predictive relevance rather than simply reflecting a general dependence on input density. We noted that this effect was more pronounced for the flash stimulus, where fewer units contributed disproportionately to performance. In contrast, in the moving ball condition, the performance remained more robust to initial removals, indicating that information is distributed across a broader neuronal population.

To further characterize the properties of the neurons prioritized by the encoder, we compared the joint distributions between the bias index and three light-response features for the overall recorded population and for the consensus sets of most-attended units in each stimulus. For every seed, we identified the top-*K* units as those whose removal reduced *R*^2^ to 0.5 in the ablation analysis; we then aggregated these lists across seeds and retained only units appearing in at least half of the seeds to yield stimulus-specific subsets of consistently attended neurons.

**Figure 6:**
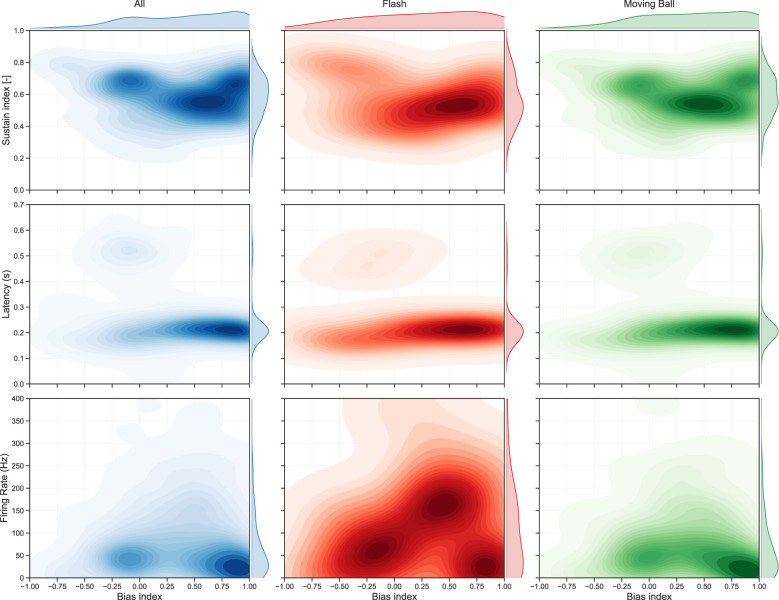
Joint distributions of physiological properties for the global population vs. high-attention units. The panels display joint kernel density estimates (KDE) of three key response features—sustain index, latency, and firing rate—plotted as a function of the bias index. The plots compare the distributions for the global population of recorded units (blue, *n* = 512) with the subsets of units most strongly attended by the model under the flash (red, *n* = 98) and moving ball (green, *n* = 230) stimuli. The attended subsets comprise unique neurons that appeared in the top-attended sets of at least four of the eight independently trained models.

Figure 6 shows the resulting two-dimensional KDE distributions, with the corresponding statistical analysis detailed in Table 4. Under the flash stimulus, we found that the most-attended neurons display a distinct physiological profile characterized by significantly higher sustain indices and elevated firing rates compared to the global population. While the KDE visualizations suggest concentration modes in the bias-feature planes, the statistical comparisons for bias index and response latency revealed that these features remain indistinguishable from the overall recorded population. For the moving ball stimulus the attended units exhibit physiological distributions that closely follow the global population across all measured features. As shown in Table 4, no significant differences were found for the bias index, sustain index, latency, or firing rate in the moving ball condition. Furthermore, a direct comparison between the two stimuli confirms that the encoder selects units with significantly different firing rate distributions depending on the visual task. Together, these trends indicate that while the model leverages specialized, high-activity neurons to decode uniform global changes like the flash, it relies on a more diverse and representative sample of the retinal population to reconstruct complex motion-rich inputs.

**Table 4:** Statistical analysis of physiological features in high-attention units.

Comparison	Bias index		Sustain index		Latency		Firing rate	
	*D*	*P*-value	*D*	*P*-value	*D*	*P*-value	*D*	*P*-value
Flash vs. All	0.142	0.476	0.238	0.041	0.127	0.617	0.248	0.029
Ball vs. All	0.048	0.830	0.047	0.847	0.072	0.361	0.039	0.955
Flash vs. Ball	0.130	0.629	0.201	0.150	0.103	0.872	0.255	0.031

The table presents a comparison of light-response features between stimulus-specific high-attention subsets and the global retinal population using the two-sample Kolmogorov-Smirnov test. Values in bold indicate *P* < 0.05.

### 3.4 Decoder attention patterns and latent-output interactions

**Figure 7:**
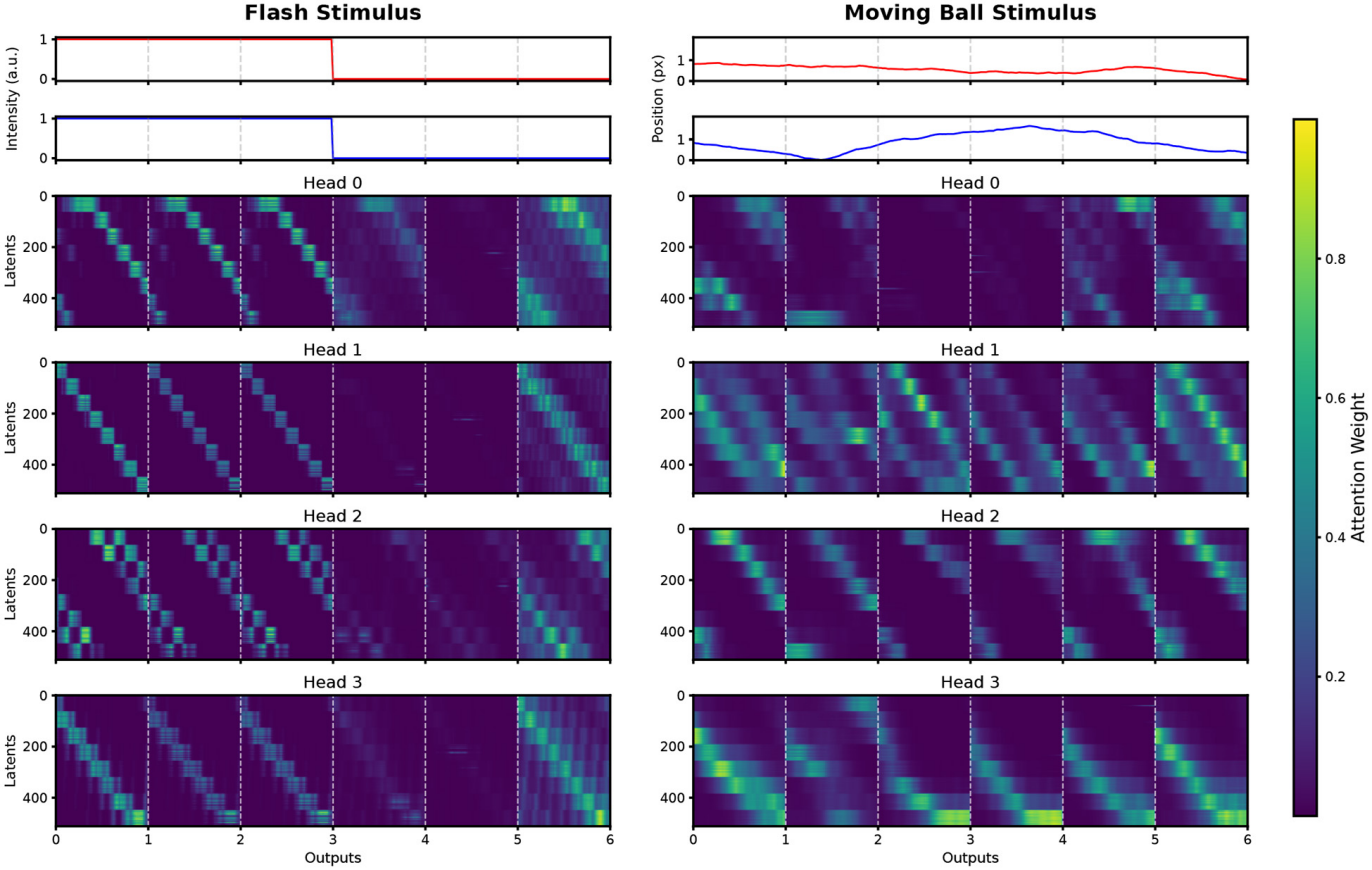
Decoder cross-attention maps linking latent representations to output predictions. The heatmaps display cross-attention patterns between latent projection space and output tokens for the two evaluated stimuli, using a representative subset of four attention heads per condition. Attention weights indicate how strongly each output token queries specific latent vectors. (Left) For the flash stimulus, characterized by purely temporal variations, attention is organized in repetitive, band-like patterns aligned with luminance changes. (Right) In contrast, for the moving ball stimulus, the attention maps exhibit a more heterogeneous and distributed structure across heads and time, reflecting the processing of combined spatial and temporal dynamics. The top panels for each column illustrate the ground-truth stimulus features: light intensity for the flash (Left) and positional coordinates for the moving ball (Right).

Finally, we analyzed the decoder's cross-attention mechanism, which links latent representations to the output sequence during stimulus reconstruction. For each stimulus type, we extracted attention matrices across all eight heads and visualized them as heatmaps with latent projection space indices on the vertical axis and output timepoints on the horizontal axis. Figure 7 presents these results alongside the stimulus trajectories for a representative subset of four attention heads per condition. In the flash condition, we observed that decoder attention is organized in repetitive, band-like patterns aligned with luminance changes.This structure suggests a uniform retrieval strategy where the model queries the latent space in synchronized intervals to track global intensity. In contrast, the moving ball condition yields more heterogeneous and distributed patterns.We noted that while some heads focus on narrow latent subsets during rapid position changes, others distribute attention more diffusely, reflecting a more complex integration of spatial and temporal dynamics.

**Figure 8:**
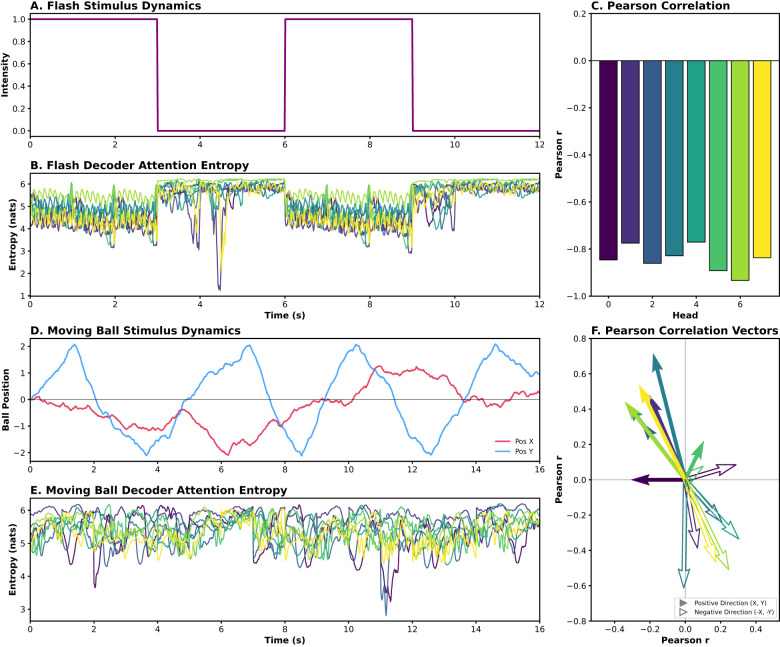
Quantitative analysis of decoder attention entropy and stimulus coupling. **(A)** Temporal dynamics of the flash stimulus light intensity. **(B)** Evolution of Shannon Entropy for the eight decoder attention heads during the flash sequence. **(C)** Pearson correlation coecients for all heads under the flash condition. **(D)** Spatiotemporal dynamics of the moving ball stimulus, showing horizontal (x) and vertical (y) positions. **(E)** Evolution of Shannon Entropy for the decoder heads during the moving ball sequence. **(F)** Pearson correlation vectors for each head. Solid arrows represent tuning toward positive direction, while hollow arrows indicate tuning toward negative direction.

To quantify the efficiency of this information retrieval, we analyzed the Shannon entropy of the decoder attention distribution. As shown in Figure 8, for the flash stimulus, all eight attention heads show entropy traces that are tightly coupled to the light intensity, resulting in consistently high negative Pearson correlation coefficients ranging from *r* = −0.77 to *r* = −0.93, confirming that the decoder increases its focus whenever a significant luminance transition occurs. In the moving ball condition, however, the relationship between attention entropy and stimulus position reveals a specialized functional organization. We observed that attention heads exhibit distinct directional tuning preferences: certain heads show strong negative correlations specifically for motion in the lower visual field (e.g., Head 3), while others are tuned to horizontal or upper-quadrant trajectories. These quantitative results indicate that the decoder does not merely track the stimulus globally; instead, it adopts a spatially selective strategy where individual heads specialize in monitoring specific regions of the visual field to reconstruct complex trajectories.

## 4 Discussion

In the present research, we evaluated the POYO architecture not only as a high-performing neural decoder but also as a potential tool for gaining insights into retinal coding. While we observed that the model consistently achieved high decoding accuracy across retinas and stimuli, our primary focus was to assess whether its internal mechanisms could provide interpretable structure that aligns with known neurophysiology.

First, the fine-tuning procedure makes the model rapidly adapt to new retinas with only a few epochs, suggesting that POYO extracts transferable latent structures that capture general principles of retinal encoding rather than merely memorizing training data. Such flexibility is critical given the inherent volatility of biological sensory systems, where fluctuations in synaptic strength and neural noise can severely limit the information transmission of static, optimal linear decoders (Sprague et al., 2015; Hendler et al., 2026).

Second, by utilizing a dynamic attention mechanism, our findings suggest that POYO's ability to rapidly align its internal representations allows it to bypass the SNR saturation typically found in volatile biological regimes, effectively mitigating the information loss imposed by such instability (Sprague et al., 2015). Furthermore, by utilizing spike-tokenization instead of the traditional temporal binning found in architectures like NDT2, POYO preserves the sub-millisecond precision necessary to decode complex spatiotemporal dynamics. This enables the identification of an invariant-like manifold of neural activity that remains robust across different retinal preparations. Consequently, our approach shows that attention-like dynamics enable flexible population coding, bridging visual attention mechanisms and overcoming the limits of fixed optimal decoding under synaptic coarse-tuning (Sprague et al., 2015; Glaser et al., 2020).

To further investigate the nature of these learned features, we examined how the model's internal priority, represented by its attention weights, aligns with known physiological response patterns. Specifically, the encoder attention analysis reveals that the model spontaneously recovers biological coding strategies by directly mapping individual spikes to latent representations. For the flash stimulus, we observed that attention appeared broadly distributed, consistent with the global and uniform nature of luminance-driven responses (Chichilnisky, 2001). The highly synchronized entropy traces observed across all attention heads support this view, indicating that the model recruits the neural population into a redundant, unified representation for uniform inputs. Although the high sensitivity of the K-S test detects minor variations in these distributions, the resulting *p*-values remain relatively close to the significance threshold, confirming the absence of strong functional divergence.

In contrast, the moving ball stimulus induced highly heterogeneous entropy profiles, marked by a profound statistical divergence between attention heads (*P* < 10^−9^). We interpret this massive statistical separation in entropy levels as evidence of a functional ‘division of labor', where the heads desynchronize to capture the diverse spatiotemporal features—such as position and motion direction—inherent to complex trajectories (Gollisch and Meister, 2010; Baden et al., 2016). Such transition from synchronized to desynchronized states demonstrates how the transformer's internal strategy dynamically adapts its computational complexity to the structure of the visual input (Vaswani et al., 2017; Ye and Pandarinath, 2021).

Specifically, we propose that this mechanism functionally emulates dynamic gain control (Demb, 2008) and lateral inhibition mechanisms that characterize retinal circuits. By de-synchronizing its attention, the model effectively suppresses global redundancy to prioritize specific, non-redundant visual features, a hallmark of efficient sensory coding. This interpretation is consistent with the findings of Nirenberg et al. (2001), who demonstrated that retinal ganglion cells act largely as independent encoders, with more than 90% of stimulus information being recoverable even when correlations are ignored. Our results suggest that by emulating these biological filtering strategies, the POYO framework spontaneously adopts a multi-channel weighting strategy that exploits this neuronal independence to resolve complex spatiotemporal dynamics. Nevertheless, we acknowledge that attention does not guarantee causal relevance, and high weights may also reflect correlation, redundancy, or shared stimulus drive (Paninski and Cunningham, 2018). Thus, although encoder attention provides interpretable signals, we maintain that its biological significance requires further validation (Duncker and Sahani, 2021; Kriegeskorte and Douglas, 2015).

To address this gap, we performed an attention-guided ablation analysis and quantified the seed-level stability of the top-ranked units across eight independent training runs. The recurrence of specific high-attention units across seeds confirms that the model consistently converges on a robust subset of informative neurons. Our results demonstrate that progressively removing units in attention rank order led to systematic and stimulus-specific performance degradation (Figure 5). In the flash stimulus, decoding performance declines rapidly and nearly monotonically, revealing a concentrated coding where a few units carry the majority of the signal (Pillow et al., 2008). For the moving ball, performance remains robust despite initial removals, consistent with a distributed and functionally diverse representation where information is shared across a broader neuronal population (Marre et al., 2015). Importantly, we emphasize that this relationship between attention and functional relevance is not trivial. Many high-performance neural decoders rely on complex internal representations where attribution scores do not necessarily correspond to causal contribution (Glaser et al., 2020; Duncker and Sahani, 2021). Here, we provided the missing causal link via the ablation analysis: units that received consistently high attention were precisely those whose removal most impaired decoding. This causal validation strengthens the case for using attention as a principled guide to neuronal relevance, allowing us to investigate whether these prioritized units share distinctive physiological properties.

The statistical comparison of light-response features between the global population and high-attention subsets reveals distinct selection profiles that depend on the visual task (Figure 6). Under the flash stimulus, the encoder's prioritization of a subset with significantly higher firing rates and a shift toward sustained temporal responses suggests a strategy aimed at maximizing total information capacity. This focus on temporal persistence allows the model to track strong global luminance modulations, exploiting the fact that higher discharge frequencies are associated with greater absolute information rates (Baden et al., 2016; Koch et al., 2004). A different strategy emerges for the moving ball stimulus, where high-attention units do not deviate from the overall population in terms of bias, sustainability, firing rate, or latency diversity. We interpret this reliance on the population's intrinsic diversity as an adaptation to the greater spatial structure and directional requirements of complex motion, where a more heterogeneous population is necessary (Fiscella et al., 2015; de Vries and Baylor, 2002). Collectively, these results indicate that the encoder allocates attention based on the spatiotemporal demands of the task by balancing the trade-off between absolute information rate and coding efficiency (Koch et al., 2004). This suggests that POYO's attention mechanism autonomously recovers the metabolic and computational principles of the vertebrate retina, shifting its selection criteria to match the information-theoretic demands of the visual input (Gollisch and Meister, 2010; Meister et al., 1995; Koch et al., 2004).

In the decoder, while the functional meaning of the learned latent vectors remains abstract, our entropy-based analysis reveals that its internal logic is surprisingly interpretable. Under the flash stimulus, we observed that decoder attention organizes into repetitive, band-like patterns where all heads redundantly track the global luminance modulation. This is confirmed by the entropy traces, which show a strong, consistent correlation with the temporal profile of the light intensity (*r* between –0.77 and –0.93). In the absence of spatial complexity, the model adopts a unified retrieval strategy, focusing its computational resources on the only available stimulus dimension.

A different regime emerges during the moving ball stimulus, where attention patterns become highly heterogeneous. Our directional decomposition reveals that these heads exhibit distinct directional tuning preferences, clearly visualized by the divergent Pearson correlation vectors in Figure 8F. The spatial orientation of these vectors demonstrates that individual heads specialize in monitoring specific regions and directions within the visual field, effectively partitioning the stimulus space. This specialized spatial organization provides a compelling functional analogy to the parallel processing streams of the vertebrate retina. Specifically, the observed “division of labor” among decoder heads mirrors the physiological organization of direction-selective ganglion cells (DSGCs). Just as DSGCs are organized into cardinal directions to encode motion vectors (Vaney et al., 2012; Fiscella et al., 2015), the POYO framework spontaneously adopts a multi-channel weighting strategy to resolve spatiotemporal complexities. This indicates that the model does not merely perform high-dimensional regression; it implicitly recovers biological coding principles to filter redundant information in favor of local motion features. While the lack of explicit physiological labels for the latents remains a challenge, these entropy-based correlation vectors offer a robust quantitative window into the logic of neural population codes.

## 5 Conclusions

We conclude that transformer-based architectures, specifically the POYO framework, do not merely achieve state-of-the-art decoding performance but spontaneously recover fundamental biological coding strategies. Our analysis of encoder-decoder dynamics reveals that the model adaptively redistributes its computational resources based on stimulus complexity, shifting from a redundant, synchronized pooling for global flashes to a highly specialized, heterogeneous recruitment of attention heads for structured motion.

By establishing a direct causal link through attention-guided ablations, we demonstrate that the model's internal prioritization is physiologically grounded: it identifies and relies on specific neural subpopulations whose removal systematically degrades decoding integrity. Furthermore, the model's ability to selectively weight units with distinct signatures, such as sustained temporal responses for luminance tracking, confirms that artificial attention can serve as a principled proxy for understanding how the retina allocates information across parallel functional channels (Baden et al., 2016; Fiscella et al., 2015).

While we acknowledge that the abstract nature of learned latents still poses challenges for full mechanistic transparency, our work demonstrates that when validated through entropy metrics and statistical hypothesis testing, these architectures offer a transparent window into the logic of neural population codes. The ability of POYO to autonomously prioritize relevant physiological features across different retinal preparations positions these models not just as decoders, but as powerful tools for automated biological discovery in large-scale neuroscience.

As further research, we propose that future progress will require integrating deep architectures with interpretability-focused approaches, including causal perturbation analyses, comparisons to receptive-field models, and frameworks that encourage biologically grounded latent structure (Bengio et al., 2019). Only by bridging predictive power with mechanistic transparency will models such as POYO advance beyond black-box decoding and contribute more directly to our understanding of retinal computation.

From the retinal coding perspective, it would be of interest to further investigate the different coding strategies implemented within each attentional encoder head, helping us elucidate the conditions that trigger either population, pairwise, or independent neural encoding (Pillow et al., 2008; Averbeck et al., 2006; Nirenberg et al., 2001).

## Data Availability

The raw data supporting the conclusions of this article will be made available by the authors, without undue reservation.
